# Probable Change of Sleep Parameters after Resection and Reconstruction Surgeries in Patients with Oral Cavity or Oropharyngeal Cancers

**DOI:** 10.1155/2021/7408497

**Published:** 2021-10-01

**Authors:** Ethan I. Huang, Shu-Yi Huang, Yu-Ching Lin, Chieh-Mo Lin, Chin-Kuo Lin, Hsuan-Keng Yeh, Chao-Min Wu

**Affiliations:** ^1^Department of Otolaryngology, Chang Gung Memorial Hospital, Chiayi 61363, Taiwan; ^2^Sleep Center of Chang Gung Memorial Hospital, Chiayi 61363, Taiwan; ^3^School of Medicine, Chang Gung University, Taoyuan 33302, Taiwan; ^4^Division of Pulmonary and Critical Care Medicine, Chang Gung Memorial Hospital, Chiayi 61363, Taiwan; ^5^Department of Nursing, Chang Gung University of Science and Technology, Chiayi 61363, Taiwan; ^6^Department of Respiratory Care, Chang Gung University of Science and Technology, Chiayi 61363, Taiwan; ^7^Graduate Institute of Clinical Medical Sciences, College of Medicine, Chang Gung University, Taoyuan 33302, Taiwan; ^8^Department of Plastic and Reconstructive Surgery, Chang Gung Memorial Hospital, Chiayi 61363, Taiwan

## Abstract

In patients of oral cavity or oropharyngeal cancers, resection of the tumor and reconstruction of the defect may reduce the framework, add a bulky flap, alter the tissue flexibility, and contribute to postoperative obstructive sleep apnea (OSA). Postoperative OSA and the potential consequences may decrease the survival rate and reduce patients' quality of life. It is unclear whether the surgery is associated with postoperative OSA. Here, we compared the polysomnographies (PSGs) before and after the surgery in 15 patients of oral cavity or oropharyngeal cancers (out of 68 patients of head and neck cancers) without a chemo- or radio-therapy. Each patient received the second PSG before the start of any indicated adjuvant therapy to prevent its interference. There were 14 men and 1 woman, with a mean age and a standard deviation (SD, same in the following) of 56.2 ± 12.8 years. There were 6 tongue cancers, 5 buccal cancers, 2 tonsil cancer, 1 lower gum cancer, and 1 trigone cancer. The results show that the surgery changed sleep parameters insignificantly in apnea-hypopnea index (AHI), mean oxyhemoglobin saturation of pulse oximetry (SpO2), minimum SpO2, mean desaturation, and desaturation index but increased mean heart rate in the patients with free flaps. These results hint that the effect of surgery on developing OSA was small in this sample, with a longer plate or a larger framework for a bulkier free flap. It needs future studies with a large sample size to generalize this first observation.

## 1. Introduction

Surgery is a solution of treating oral cavity or oropharyngeal cancers. But surgery may contribute to OSA by restricting upper airway with bulky flap [1] or prosthetic [2, 3] reconstructions. Patients of oral cavity or oropharyngeal cancers undergoing a surgery reportedly have a higher prevalence of moderate to severe postoperative OSA, compared with a nonsurgical group [4]. Few researchers reported the link between the surgical intervention and the postoperative OSA. The reported OSA associated procedures include partial glossectomy with radial forearm-free-flap reconstruction [5] and mandibulectomy without reconstruction [6]. These surgeries may reduce the framework anatomy, enlarge the content volume, or alter the tissue flexibility, and contribute to OSA.

OSA is characterized by intermittent oxygen (O2) desaturation (e.g., see [7] for a review). Studies associated the desaturation with elevate circulating epinephrine level [8–12]. Elevated serum epinephrine level contributes to elevated heart rate [8–13]. These hemodynamic changes have been linked to possible consequences such as cardiovascular disorders [14–17], metabolic syndrome, ischemic heart disease [14, 16–18], pulmonary hypertension [16, 18], congestive heart failure [16–18], cardiomyopathy [16, 18], arrhythmia [18], systemic hypertension [14], stroke [14, 16, 17, 19], and an increased mortality risk [14, 19]. Postoperative OSA and the potential consequences may not only decrease the survival rate but also reduce patients' quality of life [20]. Few (if any) researchers reported individual sleep parameters in each patient before and after the surgery, without the interference of induction or adjuvant therapies. This lack of information increases the concern of whether we are healing without hurting patients.

Here, we enrolled patients of oral cavity or oropharyngeal cancers undergoing resection and reconstruction surgeries without a preoperative chemo- or radio-therapy. Each patient underwent a PSG before the surgery and a second one before the start of any indicated adjuvant therapy to prevent its interference. We listed the variables that could be associated with development of postoperative OSA, such as tumor location, T and N stages, resection procedures, reconstruction procedures, size of the defect, and size of the flap. Then, we scatter plotted and compared each essential sleep parameter before and after the surgery, including AHI, mean SpO2, minimum SpO2, mean desaturation, desaturation index, and mean heart rate. We examined whether the sleep parameters before and after the surgery were statistically different.

## 2. Materials and Methods

From 2017 to 2019, 68 patients of head and neck cancers received a surgery by Huang with orders of PSGs. We enrolled patients that met these criteria:

- Diagnosed with oral cavity or oropharyngeal cancers
- No earlier surgery, radiotherapy, or chemotherapy for the cancer
- Receiving a resection surgery by Huang and a reconstruction surgery by Huang, Yeh, or Wu
- Pre- and postoperative PSGs were ordered, with no radio- or chemotherapy

We excluded exclude patients with these criteria:

- No or only one of the pre- and postoperative PSGs were available, in which causes included clinical schedule issue
- Dependent feeding tube that prevented a reliable PSG
- The wound or postoperative anatomy prevented a PSG
- Dependent tracheostomy

We recorded tumor location and stage according to the medical records and the 8th edition of the American Joint Committee on Cancer (AJCC) staging system [21]. The resection, reconstruction, defect size, and flap size were obtained from the operation records. The defect and flap sizes in the format of gross measurement were described by length∗width (cm^2^). Free flaps were flaps that did not belong to a neighbor structure, such as an anterolateral thigh flap or a medial sural artery perforator fasciocutaneous flap. We set the flap size of a reconstruction without a free flap to 0, including a local mucosal flap and Terudermis.

For each sleep parameter (of AHI, mean SpO2, minimum SpO2, mean desaturation, desaturation index, and mean heart rate), we scatter plotted individual pre- and postoperative data and calculated a pair *t*-test to examine the changes against no change after the surgery. The statistical significance was tested as *α* = 0.05.

We performed the statistical examinations in MATLAB 9.4.0.813654 (MathWorks, Natick, MA, USA).

### 2.1. Ethical Statements

The Institutional Review Board (IRB) of Chang Gung Medical Foundation approved this study (202001198B0) on Jul. 7, 2020.

## 3. Results

Out of the 68 patients, 15 patients of oral cavity or oropharyngeal cancers met all of the inclusion criteria and composed the sample. There were 14 men and 1 woman, with a mean age and an SD of 56.2 ± 12.8 years. There were 6 tongue cancers, 5 buccal cancers, 2 tonsil cancer, 1 lower gum cancer, and 1 trigone cancer. The mean defect size and flap size were 59.5 ± 61.8 cm^2^ and 93.7 ± 88.7 cm^2^, respectively. A postoperative PSG was followed 154 ± 212 days after the surgery.  Table 1 details the individual data.

**Figure 1:**
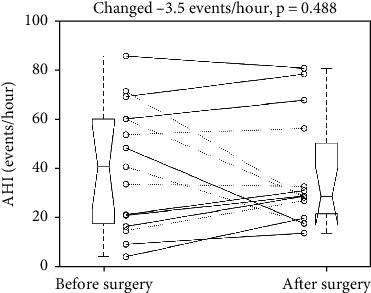
Individual changes of apnea-hypopnea index revealed an insignificant reduction after the surgery. Dotted and solid line: without and with a free flap. Each boxplot displays a five-number summary: the minimum, the maximum, the median, and the first and third quartiles.

The surgery reduced the mean AHI from 40.7 to 37.3 events/hour (*p* = 0.4884) with an SD of 25.4 and 22.2 events/hour, respectively. The mean change was -3.5 events/hour with a 95% confidence interval of -7.0 to 13.9 events/hour, which included the point of no change (0 events/hour) and showed no statistical significance.  Figure 1 illustrates the individual AHI changes and a five-number summary of pre- and postoperative AHIs. The figure showed patients undergoing reconstruction without a free flap with dotted lines for correlation. In addition to the whole sample, two pair *t*-tests to examine the changes for the free-flap ones (solid lines) and the non-free-flap ones (dotted lines) showed a nonsignificant *p* value of 0.4784 and 0.1775, respectively.

**Figure 2:**
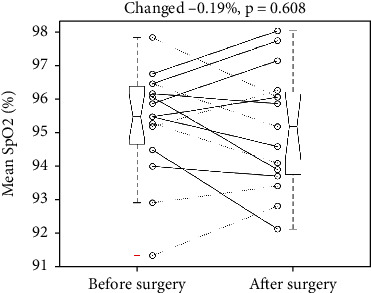
Individual changes of mean oxyhemoglobin saturation of pulse oximetry (SpO2) revealed an insignificant reduction after the surgery. Dotted and solid line: without and with a free flap. +: an outlier. Each boxplot displays a five-number summary: the minimum, the maximum, the median, and the first and third quartiles.

It reduced the mean SpO2 from 95.24 to 95.05%, *p* = 0.608. The SD was 1.64 and 1.82%, respectively. The mean change was -0.19% with a 95% confidence interval of -0.58 to 0.95%, which included the point of no change (0%) and showed no statistical significance.  Figure 2 illustrates the individual changes of mean SpO2 and a five-number summary of pre- and postoperative mean SpO2. Two pair *t*-tests to examine the changes for the free-flap ones (solid lines) and the non-free-flap ones (dotted lines) showed a nonsignificant *p* value of 0.7219 and 0.7412, respectively.

**Figure 3:**
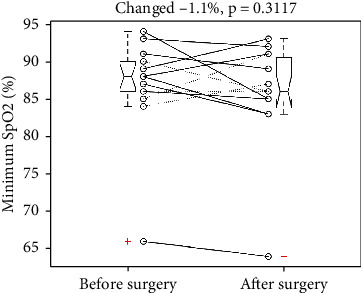
Individual changes of minimum oxyhemoglobin saturation of pulse oximetry (SpO2) revealed an insignificant reduction after the surgery. +: an outlier. Dotted and solid line: without and with a free flap. Each boxplot displays a five-number summary: the minimum, the maximum, the median, and the first and third quartiles.

It reduced the minimum SpO2 from 86.9 to 85.8%, *p* = 0.3117. The SD was 6.44 and 6.81%, respectively. The mean change was -1.1% with a 95% confidence interval of -1.11 to 3.2%, which included the point of no change (0%) and showed no statistical significance.  Figure 3 illustrates the individual changes of minimum SpO2 and a five-number summary of pre- and postoperative minimum SpO2. Two pair *t*-tests to examine the changes for the free-flap ones (solid lines) and the non-free-flap ones (dotted lines) showed a nonsignificant *p* value of 0.1894 and 0.9211, respectively.

**Figure 4:**
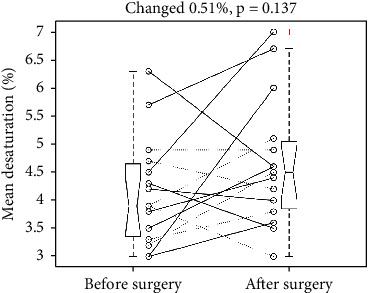
Individual changes of mean desaturation revealed an insignificant increase after the surgery. +: an outlier. Dotted and solid line: without and with a free flap. Each boxplot displays a five-number summary: the minimum, the maximum, the median, and the first and third quartiles.

It increased the mean desaturation from 4.15 to 4.66%, *p* = 0.137. The SD was 0.96 and 1.15%, respectively. The mean change was 0.51% with a 95% confidence interval of -1.21 to 0.18%, which included the point of no change (0%).  Figure 4 illustrates the individual changes of mean desaturation and a five-number summary of pre- and postoperative mean desaturation. Two pair *t*-tests to examine the changes for the free-flap ones (solid lines) and the non-free-flap ones (dotted lines) showed a nonsignificant *p* value of 0.2078 and 0.4987, respectively.

**Figure 5:**
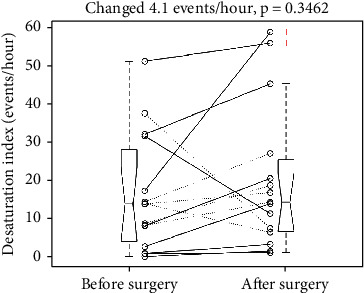
Individual change of desaturation index revealed an insignificant increase after the surgery. +: an outlier. Dotted and solid line: without and with a free flap. Each boxplot displays a five-number summary: the minimum, the maximum, the median, and the first and third quartiles.

It increased the desaturation index from 16.2 to 20.2 events/hour, *p* = 0.3462. The SD was 15.3 and 18.8 events/hour, respectively. The mean change was -4.1 events/hour with a 95% confidence interval of -13.0 to 4.9 events/hour, which included the point of no change (0 events/hour).  Figure 5 illustrates the individual changes of desaturation index and a five-number summary of pre- and postoperative desaturation index. Two pair *t*-tests to examine the changes for the free-flap ones (solid lines) and the non-free-flap ones (dotted lines) showed a nonsignificant *p* value of 0.2087 and 0.8854, respectively.

**Figure 6:**
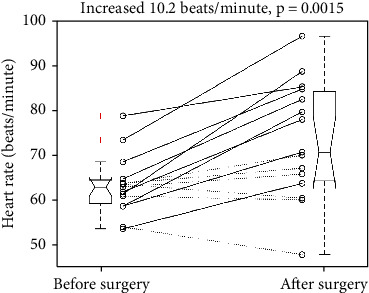
Individual mean heart rate changes revealed a significant elevation after the surgery. After the surgery, the mean heart rate increased from 63.2 ± 6.6 to 73.4 ± 13.1 beats/minute (*p* = 0.0015). +: an outlier. Each boxplot displays a five-number summary: the minimum, the maximum, the median, and the first and third quartiles.

After the surgery, the mean heart rate increased from 63.2 to 73.4 beats/minute, *p* = 0.0015. The SD was 6.6 and 13.1 beats/minute, respectively. The mean change was 10.2 beats/minute with a 95% confidence interval of 4.6 to 15.8 beats/minute.  Figure 6 illustrates the individual changes of mean heart rate and a five-number summary of pre- and postoperative heart rates. In the free-flap patients (solid lines), the surgery increased mean heart rate from 64.4 ± 7.9 to 81.1 ± 9.7 beats/minute (*p* < 0.001). The mean elevation was 16.7 beats/minute with a 95% confidence interval of 11.7 to 21.8 beats/minute. In the non-free-flap ones (dotted lines), the surgery did not change mean heart rate significantly (61.4 ± 3.79 vs. 61.9 ± 7.91 beats/minute, *p* = 0.8354).

## 4. Discussion

The results show that the resection and reconstruction surgeries did not alter sleep parameters significantly in AHI, mean SpO2, minimum SpO2, mean desaturation, and desaturation index in this sample. These sleep parameters did not differ in both free-flap and non-free-flap patients after the surgery. Plastic surgeons usually try to tailor the size of a free flap or adjust the length of metal plate to meet the defect size (e.g., see [22–24]). An experienced plastic surgeon might use a longer plate or a larger framework for a bulkier free flap. It needs to be careful when interpreting these results, including the defect and flap sizes, the sample size and locations of tumors, and the timing of the postoperative PSG. The sizes of defects and free flaps in the literature were mostly measured in the gross format of length∗width (cm^2^). The postoperative PSGs had to be conducted before an adjuvant therapy to prevent its effect and after the removal of tracheostomy or nasogastric tubes that block its enforceability. It was difficult to enroll qualified patients from the 68 patients of head and neck cancers, excluding interference factors. Our sample size is small and consists of tumors of various locations, limiting generalizability of the results. It had the unpreventable risk of unstable wound conditions and large variation of the timing. However, the results show rarely (if ever) reports of preliminary sleep data for the whole group and in both free-flap and non-free-flap patients after the surgery. Although it could be difficult, it needs future studies with large sample size and accurate volume measurement to allow the control of tumor location, flap and defect difference, and the stability of postoperative wounds, to achieve generalizability.

The only significant change was elevated mean heart rate after the surgery in the patients with free flaps. It is unclear why the change of mean heart rate was insignificant in the non-free-flap patients. Postoperative elevation of heart rate is reportedly related to catecholamine release in response to surgical stress, anemia, or pain and could be associated with vascular complication such as myocardial infarction, myocardial necrosis, or pulmonary embolism [25]. It requires future study to show a solid connection between the surgery and elevated heart rate and its underlying mechanism.

Patients with head and neck cancer had reportedly high rate of OSA (reported as 76% to 91.7%) [20, 26]. The rate is higher than the estimated prevalence in the general population (i.e., 4–24% in men and 2–9% in women [27, 28]). Fourteen out of the 15 patients (93%) had an AHI > 5 events/hour before the surgery and had OSA. There was a tendency for cancer patients with OSA to have an increase rate in postoperative morbidities (e.g., prolonged intensive care unit stay (>24 hours), the need for mechanical ventilation, and cardiopulmonary morbidities) [26]. Identification and treatment of OSA might help to avoid the postoperative morbidities and to improve quality of life for patients with oral cavity or oropharyngeal cancers [20].

## 5. Conclusions

Resection and reconstruction surgeries for oral cancers may affect the framework of anatomy, the airway space, or the flexibility. They could contribute to postoperative OSA. Postoperative OSA and the potential consequences may decrease the survival rate and reduce patients' quality of life. The results show that the surgery did not alter sleep parameters significantly in AHI, mean SpO2, minimum SpO2, mean desaturation, and desaturation index but increased mean heart rate in the patients with free flaps. The preliminary results suggest that the effect of surgery on developing OSA was small in this sample, as an experienced plastic surgeon might use a longer plate or a larger framework for a bulkier free flap. It needs future studies with a large sample size to generalize this observation.

## Figures and Tables

**Table 1:** Individual details of the enrolled patients ordered by the surgery date.

Case #	1	2	3	4	5	6	7	8	9	10	11	12	13	14	15
Age	43	34	39	70	57	61	59	61	58	74	46	44	78	63	56
Sex	M	M	M	M	M	M	M	M	F	M	M	M	M	M	M
Side	L	R	L	R	R	L	R	L	L	R	R	L	R	L	L
Surgery date	1/3/2017	9/14/2017	9/26/2017	10/31/2017	1/16/2018	3/13/2018	3/26/2018	3/27/2018	4/17/2018	5/21/2018	8/6/2018	9/18/2018	12/10/2018	12/18/2018	2/25/2019
Tumor location	Lower gum	Buccal	Tongue	Buccal	Tonsil	Tongue	Tongue	Trigone	Tonsil	Tongue	Tongue	Tongue	Buccal	Buccal	Buccal
pT stage	T2	T3	T4b	T2	T2	T2	T1	T2	T2	T2	T3	T4a	T1	T4a	T4a
pN stage	N0	N2b	N0	N0	N0	N1	N0	NA	N0	N0	N1	N3b	N1	N0	N3b
Path	MDSCC	MDSCC	WDSCC	MDSCC	HPVMSCC	MDSCC	WDSCC	WDSCC	PDSCC	WDSCC	MDSCC	MDSCC	WDSCC	MDSCC	MDSCC
Resect surgery	abc	abcd	bcde	adf	ab	ac	ab	af	ab	ab	abe	beg	ab	abce	abcd
Reconst surgery	AB	AB	AB	A	C	BD	E	GH	CE	E	A	AB	H	ABCI	AB
DS	36	28	138	20	24	36	5	25	15	15	160	144	10	180	56
FFS	72	230	175	105	0	96	0	0	0	0	176	200	0	176	176
Free flap	Y	Y	Y	Y	N	Y	N	N	N	N	Y	Y	N	Y	Y
Postop PSG	577	36	42	23	547	21	15	136	38	522	57	16	228	29	23
Postop △mHR	12.1	10.1	23.2	27.4	3.4	16.2	6.4	-3.1	2.9	-6.2	6.5	17.8	-0.9	16.3	21.1

M: male; F: female; L: left; R: right; NA: not available (no neck dissection); Path: pathology; WD: well differentiated; MD: moderately differentiated; PD: poorly differentiated; SCC: squamous cell carcinoma; HPVM: human papillomavirus-mediated; Resect: resection; Reconst: reconstruction; a: wide excision; b: same-side neck dissection; c: same-side segmental mandibulectomy; d: same-side inferior maxillectomy; e: contralateral neck dissection; f: marginal mandibulectomy; g: total glossectomy; A: anterolateral thigh flap; B: plate; C: local mucosal flap; D: tensor fascia lata myocutaneous flap; E: local tongue flap; G: buccal fat pad; H: Terudermis; I: local skin flap; J: medial sural artery perforator fasciocutaneous flap; DS: defect size (cm^2^); FFS: free-flap size (cm^2^); Postop PSG: days a postoperative polysomnography was done after the surgery; Postop △mHR: postoperative mean heart rate change (beats/minute).

## Data Availability

The data used to support the findings of this study are included within the article.
